# P-158. Knowledge of Rabies Prevention Measures for Dog Bite Incidents in the Peruvian Population: Findings from a National Survey in 2022

**DOI:** 10.1093/ofid/ofae631.363

**Published:** 2025-01-29

**Authors:** Jesus Perez-Castilla, Abraham De-Los-Rios-Pinto, Raysa M Benito-Vargas, David Soriano-Moreno, Daniel Fernandez-Guzman, Jose A Gonzales-Zamora

**Affiliations:** Universidad Nacional de San Antonio Abad del Cusco, Cusco, Cusco, Peru; Escuela Profesional de Medicina Humana, Universidad Nacional de San Antonio Abad del Cusco, Cusco, Cusco, Peru; Universidad Nacional de San Antonio Abad del Cusco, Cusco, Cusco, Peru; Unidad de Investigación Clínica y Epidemiológica, Escuela de Medicina, Universidad Peruana Unión, Lima, Lima, Peru; Escuela Profesional de Medicina Humana, Universidad Nacional de San Antonio Abad del Cusco, Cusco, Cusco, Peru; Infectious Disease Division. University of Miami, Miller School of Medicine., Miami, Florida

## Abstract

**Background:**

Rabies continues to be a significant global zoonotic threat. In the last two decades, Peru has reported nine dog-transmitted rabies cases, including a preventable death in 2023. This study aimed to assess public knowledge of rabies prevention post-dog bites, focusing on sociodemographic disparities to better tailor future interventions.

**Figure 1.**
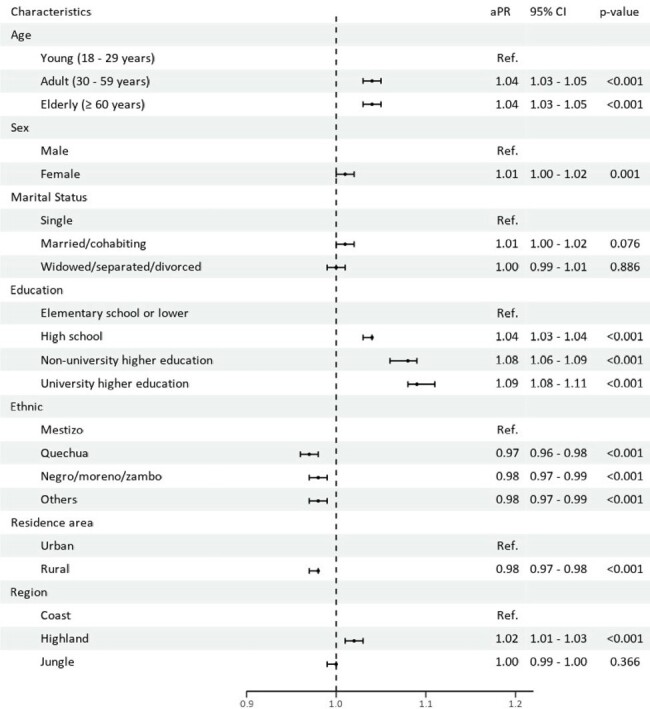
Factors associated with knowledge of the Rabies Preventive Triad of the Peruvian population, ENAPRESS 2022.

**Methods:**

Utilizing data from the 2022 National Survey of Budget Programs, targeting Peruvians aged 14 and older through a stratified two-stage sampling across both urban and rural areas, this cross-sectional study included 89,655 adults who completed interviews on the rabies prevention triad (wound washing, biting animal identification, and medical consultation). The "Survey" package in R facilitated data analysis, acknowledging the complex design. Poisson regression was employed to explore sociodemographic disparities in knowledge.

**Results:**

Out of 89,655 participants, the majority were women (53.7%), aged 30-59 (52.2%), and lived in urban areas (81.6%). The 6.5% displayed adequate knowledge of the rabies preventive triad, 45.7% indicated they would clean their wounds, 20.9% would identify the dog involved, and 86.7% would seek medical attention at a health facility. Regression models revealed better knowledge among older adults and the elderly (adjusted prevalence ratio (aPR): 1.04; 95% confidence interval (95%CI): 1.03-1.05), females (aPR: 1.01; 95% CI: 1.00-1.02), and those with higher educational levels. Highland residents showed greater knowledge than coastal ones (aPR: 1.02; 95% CI: 1.01-1.03). Conversely, Quechua ethnicity and rural residency were negatively associated with knowledge (aPR: 0.97; 95% CI: 0.96-0.98 and aPR: 0.98; 95% CI: 0.97-0.99, respectively) (Figure 1).

**Conclusion:**

Despite the high frequency of dog bites in Peru, knowledge of rabies prevention is very low. Although limited by potential recall bias and its non-causal inference capacity, the study's strengths lie in its national scope and reliable data collection. The results underscore the urgency of targeted educational campaigns and policy enhancement in high-risk areas to boost community health and rabies prevention.

**Disclosures:**

**All Authors**: No reported disclosures

